# Spontaneous Paraclinoid Carotid Artery Bleeding Presenting as Massive Epistaxis: A Case Report and Literature Review

**DOI:** 10.7759/cureus.88900

**Published:** 2025-07-28

**Authors:** Rana Alahmadi, Ghassan Bin Lajdam, Saad Alqarni, Mehwish Khattak, Saeed Alsharif

**Affiliations:** 1 Medicine, King Saud Bin Abdulaziz University for Health Sciences College of Medicine, Jeddah, SAU; 2 Otolaryngology - Head and Neck Surgery, King Abdullah Medical City, Jeddah, SAU; 3 Otolaryngology - Head and Neck Surgery, King Fahad Armed Forces Hospital, Jeddah, SAU; 4 Internal Medicine, King Fahad Armed Forces Hospital, Jeddah, SAU

**Keywords:** aspergillus, case report, endovascular embolization, epistaxis, fungal pseudoaneurysm, invasive fungal sinusitis, non-traumatic aneurysm, supraclinoid internal carotid artery

## Abstract

Fungal pseudoaneurysms of the internal carotid artery (ICA) are rare, life-threatening complications of invasive fungal sinusitis, particularly in immunocompromised patients. These pseudoaneurysms typically occur in the cavernous segment due to its proximity to the sphenoid sinus and often present with massive epistaxis or neurological deficits. The supraclinoid ICA segment is a rare location for these aneurysms and carries risks of both intracranial and sinonasal hemorrhage. This case report presents an 82-year-old immunocompromised male who experienced sudden massive epistaxis and hemoptysis. Initial CT imaging showed left sphenoid sinus opacification and bony erosion near the paraclinoid ICA, while endoscopy confirmed fungal growth identified as *Aspergillus*. After surgical debridement, the patient suffered recurrent hemorrhage, prompting CT angiography that revealed a pseudoaneurysm in the supraclinoid segment of the left ICA. Balloon occlusion testing confirmed adequate collateral flow, facilitating endovascular coil embolization. Although initial bleeding was managed, the patient later developed right hemiparesis and ultimately succumbed to complications, including myocardial infarction and aspiration pneumonia. This case underscores the importance of early recognition, urgent vascular imaging, and prompt multidisciplinary intervention in managing this critical condition.

## Introduction

Fungal or mycotic aneurysms of the internal carotid artery (ICA) are exceedingly rare, typically arising as a complication of invasive fungal sinusitis in immunocompromised patients [1,2]. *Aspergillus* species is the most commonly implicated pathogen (followed by *Mucor* and *Candida*), invading and weakening the arterial wall to form pseudoaneurysms​ [1]. These infected aneurysms have extremely fragile walls and a strong propensity to rupture, making them highly dangerous lesions.

Pseudoaneurysms develop as a result of injury to the vascular wall, which leads to the formation of an encapsulated hematoma that connects to the ruptured artery. It may vary according to several factors, including the status of the rupture, the anatomical location, and the dimensions of the pseudoaneurysm [3]. Epistaxis is often the first and most dramatic manifestation of a ruptured ICA pseudoaneurysm in this context. Massive nasal hemorrhage from a carotid aneurysm is a well-recognized catastrophic event, frequently associated with high mortality​ [2,4]. Indeed, in reported fungal ICA aneurysm cases, severe epistaxis has served as a sentinel sign of the aneurysm, and outcomes are often fatal without prompt intervention​ [4]. Even a seemingly minor “sentinel” nosebleed in a patient with invasive fungal sinusitis may precede a life-threatening carotid hemorrhage. This contrasts with the typical benign causes of epistaxis, highlighting the need for a high index of suspicion when nosebleeds occur in high-risk patients.

Anatomically, most fungal ICA aneurysms reported in the literature involve the cavernous segment of the carotid artery, owing to its proximity to the sphenoid sinus, a frequent site of invasive fungal infection​ [1]. Direct extension of a sinus fungal infection into the cavernous ICA wall is a key pathogenic mechanism in these cases. In contrast, more distal intracranial segments (beyond the cavernous sinus) are rarely involved except via hematogenous spread or extensive local invasion. Involvement of the supraclinoid (intradural) ICA segment is thus exceedingly uncommon in the fungal context, making the present case noteworthy for its atypical location. An aneurysm in this supraclinoid location poses a dual threat. This is because it can bleed into the nasal cavity if it erodes into adjacent sinus spaces, and it also carries the risk of subarachnoid hemorrhage due to its intracranial position [5].

Given the rarity and often fatal outcomes of fungal ICA aneurysms, early diagnosis is crucial yet challenging. This case report emphasizes the need for clinicians to consider vascular lesions, such as pseudoaneurysms, in patients presenting with epistaxis, particularly those with fungal sinus disease or immunocompromised conditions. We present a unique case of a fungal pseudoaneurysm of the supraclinoid ICA, which caused massive epistaxis.

## Case presentation

An 82-year-old male presented to the emergency department with sudden-onset massive epistaxis, hemoptysis, and left-sided headache. He reported coughing up approximately two cups of blood with clots. His medical history included Hepatitis B virus infection, ischemic heart disease, prostate cancer (previously treated with radiation and hormonal therapy), and heart failure with reduced ejection fraction (45%). He had undergone coronary artery bypass grafting 35 years prior and percutaneous coronary intervention of the left anterior descending artery 6 years prior. His medication regimen at the time of presentation included tranexamic acid, pantoprazole, isosorbide dinitrate, tenofovir, atenolol, and enzalutamide.

**Figure 1:**
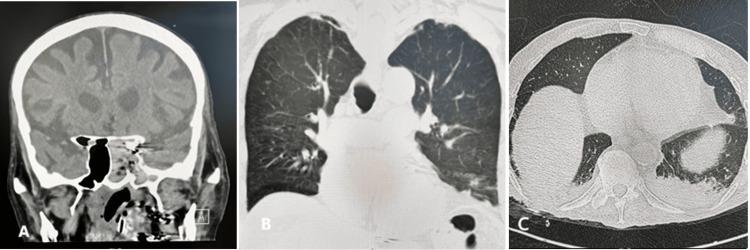
A: CT image showing opacification of the left sphenoid sinus with erosion of the clivus and marked thinning of the posterior sphenoid wall adjacent to the paraclinoid segment of the left internal carotid artery. B: Coronal chest CT revealing mild bilateral bronchiectasis. C: Axial chest CT showing bilateral pleural effusions.

Initial diagnostic evaluations, including computed tomography (CT), aimed to identify the source of bleeding. High-resolution chest CT demonstrated mild bronchiectasis and bilateral pleural effusion. CT imaging of the sinuses showed opacification of the left sphenoid sinus with erosion of the clivus and marked thinning of the posterior sphenoid wall adjacent to the paraclinoid segment of the left ICA (Figures 1A-1C). During nasopharyngoscopy, blood pooling was noted from the left sphenoid sinus, prompting placement of a left posterior nasal pack.

**Figure 2:**
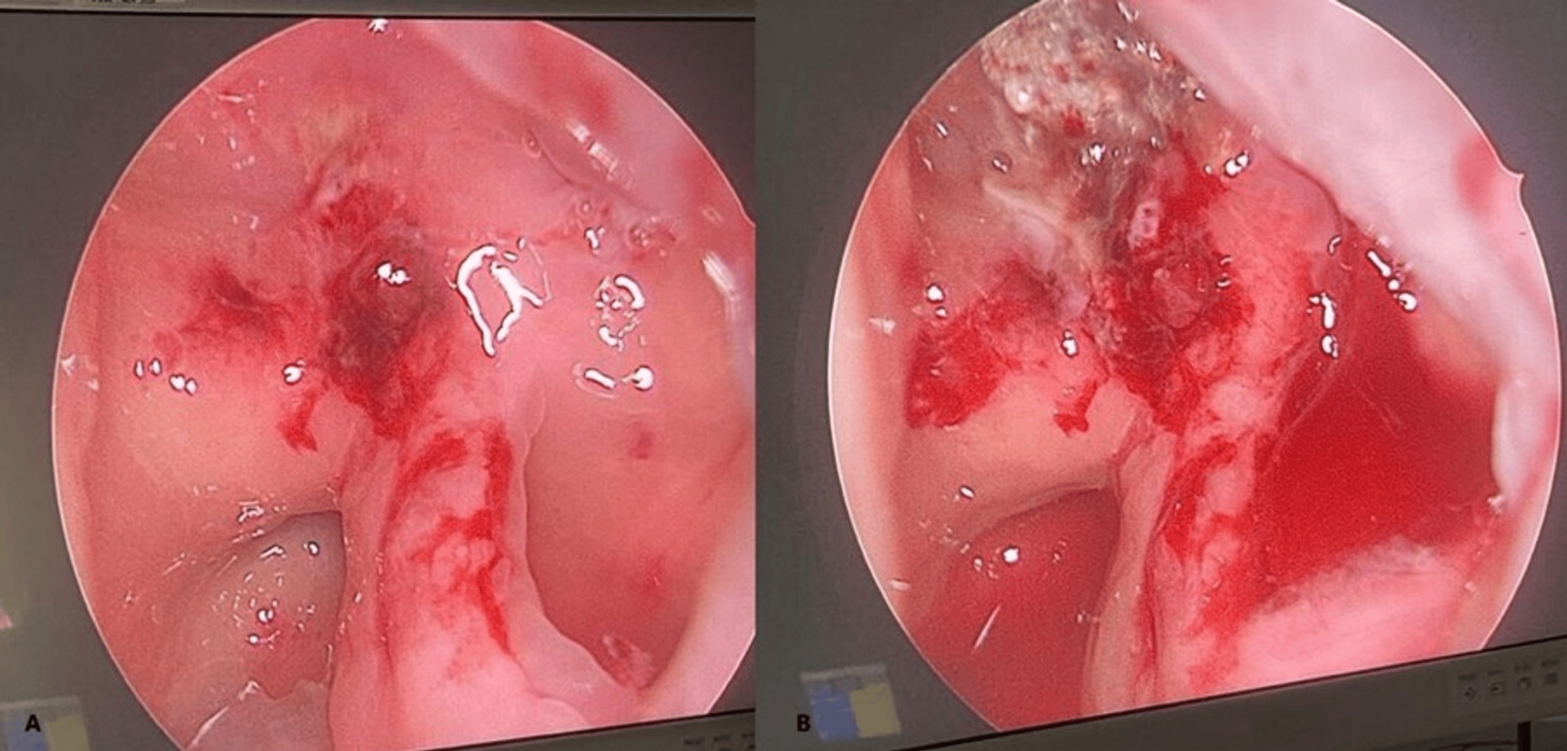
Endoscopic view showing fungal growth attached to the posterior sphenoid sinus wall adjacent to the ICA aneurysm (A); appearance of the ICA aneurysm and sphenoid sinus wall after removal of the fungal growth (B) ICA: internal carotid artery

After stabilization, one week later, the patient was taken to the operating room for surgical exploration and removal of the nasal pack. Endoscopic examination revealed two crests resembling a fungal infestation covering a dehiscent area of the posterior sphenoid sinus wall (Figure 2A). The crests were removed and sent for histopathological examination. No active bleeding was noted during the process (Figure 2B). Multiple absorbable packs were inserted into the sphenoid sinus, followed by a prophylactic left posterior nasal pack. Histopathological analysis of the crests later confirmed *Aspergillus* infection.

**Figure 3:**
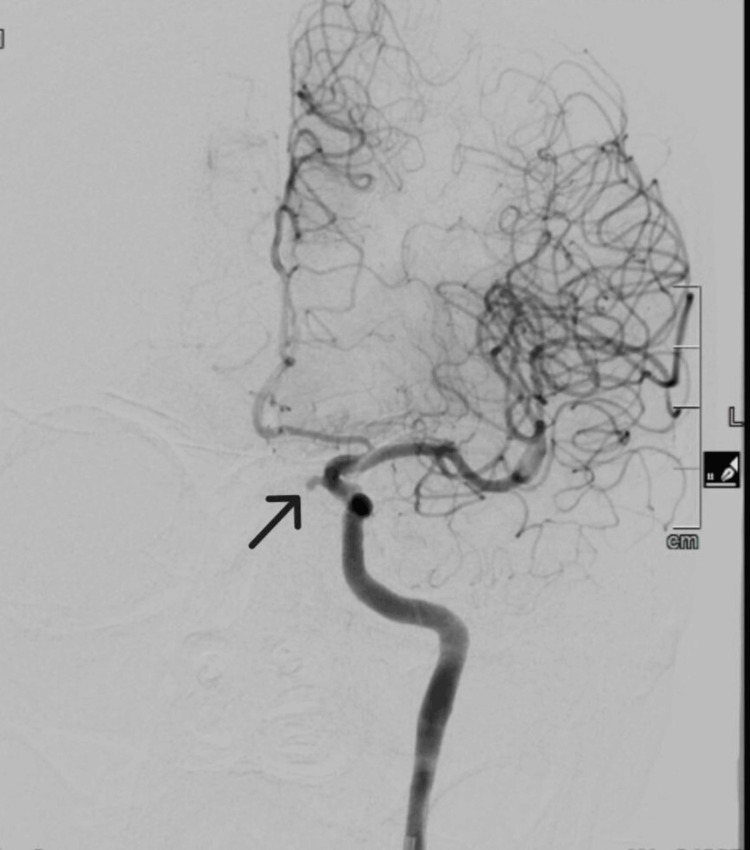
Coronal CT angiography before coiling, demonstrating a supraclinoid segment ICA aneurysm (arrow) ICA: internal carotid artery

Three days postoperatively, upon left posterior nasal pack removal in the clinic, the patient experienced a recurrent massive episode of epistaxis with an estimated blood loss of approximately 500 mL. Bilateral nasal packing was inserted with inflated balloons, and the patient was rushed to the intensive care unit (ICU) for intubation and hemodynamic stabilization. Emergent CT angiography revealed a pseudoaneurysm in the supraclinoid segment of the left ICA (Figure 3). Subsequent balloon occlusion testing of the left ICA suggested adequate cross-over circulation from the right ICA to the left cerebral hemisphere.

**Figure 4:**
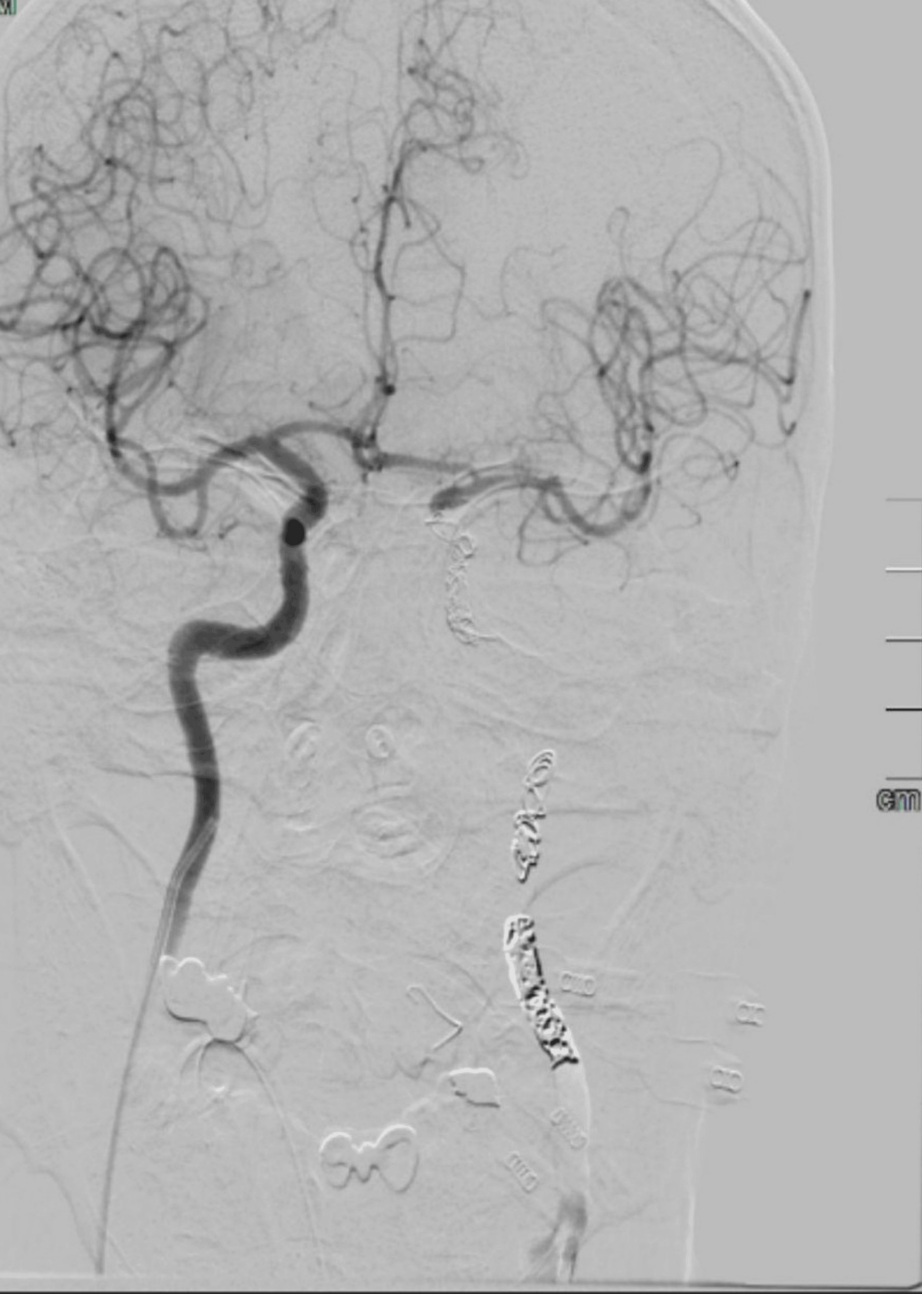
Coronal digital subtraction angiography following coil embolization, demonstrating multilevel ICA coiling with obliteration of intra-aneurysmal blood flow ICA: internal carotid artery

After multidisciplinary discussions involving radiology, neurosurgery, and infectious disease specialists, definitive endovascular management with coil embolization of the left ICA was performed successfully (Figure 4). On the following day, the nasal packs were removed, and the left sphenoid sinus was examined under general anesthesia. The examination confirmed no active bleeding; however, the posterior sphenoid wall appeared fragile and demonstrated pulsation. Two left nasal packs were inserted for maintenance, with planned removal for a later date. The patient tolerated both procedures well initially and was transferred back to the ICU. Over the subsequent period, the patient's left nasal packs were maintained and monitored daily, and no further episodes of epistaxis were observed. However, right hemiparesis was noted 24 hours after the procedure, indicative of a potential ischemic event. Despite continued intensive supportive management, further complications, including a non-ST-segment elevation myocardial infarction (NSTEMI) and aspiration pneumonia, ensued. Unfortunately, the patient's clinical condition progressively deteriorated, and he ultimately succumbed to these complications.

## Discussion

Fungal pseudoaneurysms of the ICA are rare and perilous complications of invasive sinus infections [6]. They primarily affect immunocompromised individuals, especially those with uncontrolled diabetes or neutropenia, in the context of aggressive fungal sinusitis such as mucormycosis or aspergillosis​ [7]. Although our patient did not exhibit classic immunosuppressive factors, such as diabetes, neutropenia, or corticosteroid use, he had comorbidities, including ischemic cardiomyopathy, a history of prostate cancer, and chronic hepatitis B, which may have impaired immune function. Age and chronic illness may have also contributed to vascular invasion, as fungal hyphae often infiltrate the vessel wall from the adjacent sphenoid sinus or skull base, causing necrosis and weakening​ [1]. An ICA pseudoaneurysm can remain silent until rupture. Massive epistaxis, though uncommon, is reported in approximately 9% of cases​ [7] and is often a sign of arterial rupture​ [2,8]. Rupture-related epistaxis is so rare that only a few cases are documented. This rarity, along with benign causes of epistaxis, often delays diagnosis, potentially resulting in disastrous consequences. Our case underscores that recurrent epistaxis in high-risk patients must raise suspicion for a carotid pseudoaneurysm, even without a prior sinus infection.

Anatomically, most fungal ICA pseudoaneurysms affect the petrous or cavernous segments near the sphenoid and cavernous sinuses. Cavernous ICA pseudoaneurysms are extradural and typically rupture into the sphenoid sinus or form carotid-cavernous fistulae, causing epistaxis or cranial neuropathies, but not subarachnoid hemorrhage​ [9]. In contrast, intradural pseudoaneurysms in the supraclinoid ICA are rare but particularly dangerous. Once infection spreads intradurally, rupture may lead to subarachnoid hemorrhage or stroke. Only two such supraclinoid ICA mycotic aneurysms have been reported, presenting as stroke [10] or subarachnoid hemorrhage [5], not epistaxis. Therefore, while cavernous ICA lesions often present with ENT-related warning signs, supraclinoid involvement may lead to fatal intracranial events without any preceding symptoms. Interestingly, all patients who presented with left-sided aneurysmal changes ultimately succumbed to their condition.

This case adds to the limited literature on ICA pseudoaneurysms with epistaxis. In the majority of reported cases, fungal sinusitis is recognized before aneurysm diagnosis. For example, Diyora et al. ​ described a diabetic male with mucormycosis, treated with antifungals and debridement, who later developed massive epistaxis [11]. Imaging revealed a pseudoaneurysm of the petrous ICA eroding into the sphenoid sinus. In contrast, our patient had no known history of sinusitis or prior fungal diagnosis or fungal diagnosis; fungal etiology became apparent only after admission. This illustrates a key diagnostic challenge: a fungal pseudoaneurysm may be the first sign of an occult fungal infection. Clinicians must consider underlying infection in patients with unexplained massive epistaxis, particularly if risk factors, such as immunosuppression, are present. Early ENT evaluation and urgent imaging (CTA or angiography) are critical.

Management strategies vary but aim to prevent re-rupture and control infection. Common approaches combine systemic antifungals with endovascular or open surgical interventions. Endovascular parent artery occlusion (using coils or balloons) is often effective and has been successfully used in several cases ​[7]. In our case, a supraclinoid ICA pseudoaneurysm was treated with endovascular coil embolization and parent artery sacrifice. This procedure successfully excluded the aneurysm from the cerebral circulation and achieved hemostasis. Such a sacrifice is feasible if the circle of Willis provides adequate collateral flow, often confirmed with a balloon occlusion test.

However, not all patients tolerate ICA sacrifice. When collateral flow is inadequate, abrupt occlusion risks ischemic stroke. In these cases, revascularization bypass surgery is vital. Diyora et al. reported successful use of a “double bypass” - superficial temporal artery to middle cerebral artery (STA-MCA) plus a high-flow internal carotid artery to internal carotid artery (ECA-ICA) graft - followed by cervical ICA ligation [11]. This complex but lifesaving approach preserved brain perfusion while excluding the aneurysm.

Endovascular reconstructive techniques that preserve the parent artery, such as stent-assisted coiling or flow-diverting stents, have been successful in some cases. Sano et al. described stent-assisted coiling of a cavernous ICA *Aspergillus* aneurysm, achieving hemostasis and vessel preservation [1]. Another case of COVID-19-associated mucormycosis was managed with a Pipeline™ flow diverter (Medtronic, Dublin, Ireland), which led to aneurysm thrombosis while preserving the ICA ​[12]. These techniques are promising in extradural lesions where device infection risk is low and vessel preservation is critical. However, their downside risk of infection and need for long-term antiplatelets must be weighed carefully. Deconstructive strategies are often favored in mucormycosis due to its rapid vascular invasion. One study noted an 84% ICA occlusion rate in *Mucor *cases vs. 46% in *Aspergillus*, suggesting earlier rupture or thrombosis in mucormycosis​ [7]. Evidence suggests that *Mucor* infections tend to cause early thrombosis or rupture of the ICA. One study reported an 84% ICA occlusion rate with *Mucor* infections compared to 46% with *Aspergillus*, whereas *Aspergillus* infections more commonly lead to true aneurysm formation and subsequent subarachnoid hemorrhage [7]. Thus, parent vessel sacrifice may be safer in mucormycosis, while reconstructive approaches might be more appropriate in *Aspergillus*.

The natural history of these lesions is exceedingly poor. Without treatment, recurrent hemorrhage is very likely and carries a mortality rate well above 50% [7,13]. Our patient had already experienced a sentinel epistaxis prior to admission, and literature suggests that such warning bleeds often presage a catastrophic rebleed if the pseudoaneurysm remains untreated​ [12]. Thus, early recognition and timely occlusion of the aneurysm are paramount. Equally important is concurrent management of the invasive fungal infection. A multidisciplinary team involving neurosurgery, interventional neuroradiology, otolaryngology, and infectious disease is required to address all facets of care. Surgical debridement of necrotic sinus tissue (if feasible) along with high-dose systemic antifungals should be initiated as soon as possible, in parallel with measures to secure the aneurysm. In practice, the sequence of interventions may need to be tailored to the patient’s stability. In acute hemorrhage scenarios, securing the carotid artery (endovascularly or surgically) takes priority. In more stable cases, sinus debridement may precede vascular exclusion.

This case and prior reports highlight several key lessons. First, invasive fungal sinusitis involving the ICA progresses rapidly; delays significantly worsen prognosis [7,14]. Though invasive fungal carotiditis carries high mortality, early and aggressive treatment has been shown to improve outcomes [1,11]. Second, recurrent epistaxis in patients with risk factors, such as diabetes, immunosuppression, or recent sinus infection, must be seen as a critical warning sign for possible carotid artery erosion [15]. As demonstrated, such bleeding necessitates prompt imaging (CTA, MRA, or angiography) to identify occult pseudoaneurysms before catastrophic hemorrhage occurs. Additionally, effective management demands rapid, multidisciplinary coordination. Nasal endoscopy may reveal necrotic tissue or a carotid bulge in the sphenoid region, indicating potential carotid involvement. Treatment must balance aneurysm exclusion with the preservation of cerebral perfusion. Targeted antifungal therapy, liposomal amphotericin B for *Mucor* or voriconazole for *Aspergillus*, is crucial, along with appropriate adjunctive therapies like hyperbaric oxygen. Finally, long-term follow-up is essential. Even after successful repairs, patients need monitoring for recurrence, new aneurysm formation, and persistent infection. Serial angiographic imaging may be warranted, as delayed pseudoaneurysm recurrence has been documented [16].

Table 1 presents the literature review conducted during this study.

**Table 1:** Fungal ICA pseudoaneurysms presenting with epistaxis: a literature review ICA: internal carotid artery; EF: ejection fraction; CTA: computed tomography angiography; NSTEMI: non-ST-segment elevation myocardial infarction; HTN: hypertension; ESRD: end-stage renal disease; GIST: gastrointestinal stromal tumor; CAD: coronary artery disease; STA-MCA: superficial temporal artery to middle cerebral artery; EC-IC: extracranial-intracranial

Author (Year)	Age/Sex	Underlying Conditions (Immunosuppression)	Fungus (Species)	Aneurysm Site	Aneurysm Condition	Treatment	Outcome
Current Case	82/M	Hepatitis B, ischemic heart disease, prostate cancer (radiation + hormonal therapy), heart failure (EF 45%)	*Aspergillus spp.* (confirmed on histopathology	Left ICA (supraclinoid segment)	Ruptured pseudoaneurysm (massive epistaxis, confirmed on CTA)	Endovascular coil embolization of the left ICA	Death - right hemiparesis, NSTEMI, aspiration pneumonia
Jao et al. (2011) [4]	76/M	HTN, well-controlled diabetes	*Aspergillus spp. *(identified on pathology and culture)	Left ICA (cavernous segment)	Ruptured pseudoaneurysm (thin-walled, wide-neck; massive epistaxis)	Endovascular reconstruction – flow-diversion stents + coiling	Survived – no rebleed
Little et al. (2023) [7]	77/F	ESRD, Type II DM	*Aspergillus flavus* (identified on culture from the mastoid)	Left ICA	Ruptured ICA mycotic aneurysm with acute-onset epistaxis post-treatment	Isavuconazole (37d); tympano-mastoidectomy; ICA embolization (vessel sacrifice)	Death – 150 days after presentation
Rostamihosseinkhani et al. (2022) [14]	54/M	GIST on imatinib, diabetes, COVID-19, steroids	*Mucorales* (confirmed via a nasal turbinate biopsy)	Left ICA (cavernous and supraclinoid portions)	Ruptured fusiform pseudoaneurysm (15×8×9 mm); ICA occlusion; infarcts	Amphotericin B, FESS, hemicraniotomy	Death – infarction, vasogenic edema
Sano et al. (2022) [1]	76/M	CAD, ICA stent, HTN	*Aspergillus* spp. (identified on pathology and culture)	Right ICA (into the sphenoid sinus)	Ruptured pseudoaneurysm (irregular/saccular; epistaxis, shock)	Stent-assisted coiling (preserved ICA)	Survived – no deficit, no rebleed (12-mo f/u)
Takaishi et al. (2023) [8]	63/M	None reported	*Aspergillus* spp. (identified on pathology and clinical imaging)	Left cavernous ICA	Ruptured pseudoaneurysm (massive epistaxis)	Endovascular ICA occlusion + antifungals	Survived – no deficits
Seshadri et al. (2023) – Case 1 [12]	64/M	Diabetes, post-COVID mucormycosis	*Mucorales* (confirmed via biopsy)	Left cavernous ICA	Ruptured pseudoaneurysm (massive epistaxis with syncope)	Flow-diverting stent + amphotericin B	Survived – no recurrence (8-mo f/u)
Seshadri et al. (2023) – Case 2 [12]	51/M	Diabetes, post-COVID mucormycosis	*Mucorales* (confirmed via biopsy)	Left cavernous ICA	Ruptured pseudoaneurysm (recurrent epistaxis, trigeminal pain)	Overlapping stents + antifungals	Survived – neurologically stable (1-yr f/u)
Al-Domaidat & Jawad (2024) [2]	33/F	Asthma; AFRS	Allergic fungal sinusitis (species not specified; presumed fungal)	Right petrous ICA	Ruptured pseudoaneurysm (massive, delayed epistaxis 13d post-op)	Endovascular ICA coiling	Survived – intact at 10-yr f/u
Diyora et al. (2024) [11]	43/M	Uncontrolled diabetes; invasive fungal sinusitis	*Mucorales* (zygomycetes species; identified on culture)	Left petrous ICA	Ruptured pseudoaneurysm (massive epistaxis; bony erosion)	Double bypass (STA-MCA + EC-IC) + ICA ligation	Survived – no recurrence at 6-mo f/u

## Conclusions

Fungal ICA pseudoaneurysms are a rare but deadly complication of invasive fungal sinusitis. They can present as massive epistaxis, signaling potential carotid rupture. This case underscores the need for urgent vascular imaging and a high index of suspicion in patients with severe epistaxis and suspected fungal infections. Immediate treatment, including antifungal therapy, hemodynamic stabilization, and aneurysm occlusion, is crucial. Although all methods carry risks, delays can be fatal. Early multidisciplinary coordination is key to improving outcomes in this serious vascular emergency.
